# Telehealth for amyotrophic lateral sclerosis in a multidisciplinary service in a Brazilian reference center

**DOI:** 10.1055/s-0043-1768161

**Published:** 2023-05-31

**Authors:** Emanuela Coriolano Fidelix, Glauciane Costa Santana, Daniele Montenegro da Silva Barros, Mário Emílio Teixeira Dourado Junior

**Affiliations:** 1 Universidade Federal do Rio Grande do Norte, Departamento de Medicina Integrada, Natal RN, Brazil.; 2 Universidade Federal do Rio Grande do Norte, Laboratório de Inovação Tecnológica em Saúde, Natal RN, Brazil.

**Keywords:** Amyotrophic Lateral Sclerosis, Motor Neuron Disease, Telemedicine, Esclerose Amiotrófica Lateral, Doença do Neurônio Motor, Telemedicina

## Abstract

**Background** Telehealth has been used in the treatment of different diseases, and it has been shown to provide benefits for patients with amyotrophic lateral sclerosis (ALS). Due to the social distancing measures put into effect during the coronavirus disease 2019 (COVID-19) pandemic, there was an urgent need for telehealth to ensure the provision of healthcare.

**Objective** To evaluate the feasibility of telehealth for the provision of multidisciplinary ALS care, and to assess its acceptability among patients and caregivers.

**Methods** We conducted a retrospective cohort study in which multidisciplinary evaluations were performed using the Teleconsulta platform. The patients included had ALS and at least one in-person clinical evaluation. The patients and the caregivers answered satisfaction questionnaires.

**Results** The sample was composed of 46 patients, 32 male and 14 female subjects. The average distance from their residences to the reference services was of 115 km. Respiratory adjustment was the most addressed topic.

**Conclusion** The strategy is viable and well accepted in terms of satisfaction. It was even more positive for patients in advanced stages of the disease or for those living far from the referral center.

## Resumo

**Antecedentes** A telessaúde tem sido utilizada no tratamento de diferentes doenças, e demonstrou-se que ela traz benefícios para pacientes com esclerose lateral amiotrófica (ELA). Devido às medidas de distanciamento social postas em prática durante a pandemia de doença do coronavírus 2019 ( *coronavirus disease 2019* , COVID-19, em inglês), houve uma necessidade urgente de se usar a telessaúde para garantir a provisão dos cuidados de saúde.

**Objetivo** Avaliar a viabilidade da telessaúde para a prestação de cuidados multidisciplinares na ELA, e verificar a sua aceitabilidade entre os pacientes e os cuidadores.

**Métodos** Realizou-se um estudo de coorte retrospectivo, com avaliações multidisciplinares realizadas por meio da plataforma Teleconsulta. Os pacientes incluídos apresentavam ELA, e já haviam passado por pelo menos uma avaliação clínica presencial. Os pacientes e os cuidadores responderam a questionários de satisfação.

**Resultados** A amostra continha 46 pacientes, 32 do sexo masculino e 14 do sexo feminino. A distância média de suas residências ao serviço de referência era de 115 km. O ajuste respiratório foi o tema mais abordado.

**Conclusão** A estratégia é viável e bem-aceita em termos de satisfação. Foi ainda mais positiva para os pacientes com doença avançada ou residentes em uma cidade distante do centro de referência.

## INTRODUCTION

Technology has been used in healthcare for several years through different tools (such as phone and video calls and sending digital documents), 1 and the terms *telemedicine* and *telehealth* emerged from it. Telemedicine uses technological tools to provide healthcare as well as the exchange of information between the physician and the patient, while telehealth is a broader concept involving support groups, health professionals, and information. 2 3

The telestroke was one of the first telehealth services used to perform remote clinical evaluations and make therapeutic decisions. 4 5 Telehealth has also been used to follow up patients with other conditions, 6 7 8 9 10 and it has been shown to provide benefits for patients with neuromuscular diseases, such as amyotrophic lateral sclerosis (ALS). 2 11 12

Patients with ALS and their caregivers deal with several issues as the disease progresses. The treatment for ALS is based on controlling symptoms and improving quality of life and survival, since it currently has no cure. 13 Thus, a multidisciplinary team must regularly monitor these patients through visits to increase therapeutic adherence and survival. 14 The multidisciplinary team is composed of different health professionals with expertise in ALS, like neurologists, pneumologists, nurses, dieticians, and other professionals. During the coronavirus disease 2019 (COVID-19) pandemic, social distancing hampered this approach, and telehealth was used to enhance access to specialized care for patients with ALS, regardless of geographic distance. 15 16

Therefore, this study aimed to evaluate the viability of teleconsultations for patients with ALS and the level of satisfaction of patients and caregivers at a referral center in northeastern Brazil.

## METHODS

### Study design

The present was a retrospective cohort study with ALS patients treated at the Motor Neuron Diseases outpatient clinic of Hospital Universitário Onofre Lopes, at Universidade Federal do Rio Grande do Norte (HUOL/UFRN), from March 2020 to March 2021.

### Patients

We included patients diagnosed with ALS according to the El Escorial criteria with at least one in-person consultation. Published in 1994, the criteria include categories such as suspected, possible, probable, and definite. Cases that presented during the COVID-19 pandemic were evaluated in person before the follow-up through teleconsultation. Patients without internet access or who refused to participate in teleconsultations were excluded from the study. Teleconsultation was offered to all 71 patients regardless of the stage of the disease. Caregivers and patients could contact the group coordinator by phone or text message to schedule a teleconsultation. In total, 46 (64.7%) patients participated in the teleconsultations.

### Protocol

A member of the multidisciplinary team contacted the patients through phone calls, usually one day before the teleconsultations, to apply a brief screening questionnaire. Then, synchronous teleconsultations were conducted. 9 The service's multidisciplinary team was composed of a neurologist with specialist expertise in ALS, motor and respiratory physiotherapists, a speech and swallow therapist, a dietician, and a psychologist. A shared virtual room was used, and all professionals were able to view the ongoing evaluation. Each visit takes between 1 and 2 hours.

Caregivers were invited to the teleconsultations when needed to help the patient communicate with the multidisciplinary team and clarify possible doubts and demands. The revised ALS Functional Rating Scale (ALSFRS-R) 17 and the King's Staging 18 were applied during the teleconsultations. We also collected data on symptoms, use of medication, general care, acute complications, equipment use, and results of the exams.

The patients were divided into 2 groups according to the ALSFRS-R score (≥ 39 and < 39) and ranked according to the rate of progression, calculated through the following equation: 48 ‒ ALSFRS-R score at diagnosis/duration from onset of symptoms to diagnosis (in months). Rate of progression was classified as fast (> 1), intermediate (0.5 to 1.0), or slow (< 0.5). 19

### Technological tool (teleconsulta platform)

The Teleconsulta platform was developed by the information technology team of the Laboratory of Technological Innovation in Health at HUOL/UFRN. It is a private virtual consultation room for health professionals and patients, accessed using a smartphone or computer with internet access. When the patients could not access this platform, the teleconsultation occurred using the Google Meet platform.

### Assessment of the level of satisfaction

Two questionnaires were applied to assess the level of satisfaction after each teleconsultation using a Likert scale (one for the patient and the other for the caregiver). 20

### Ethical considerations

The protocol for the present study was reviewed and approved by the Ethics Review Board of UFRN (under numbers CEP HUOL/UFRN 4.152.889).

## RESULTS

The spinal-onset classic phenotype was the most frequent among ALS patients. The probable (35%) and definite (30%) categories of the El Escorial criteria and stage 4b (59%) on the King's Staging were predominant among the studied patients.

The ALSFRS-R was applied during all evaluations, except to patients evaluated in less than three months. The patients were also investigated for signs of hypoventilation, weight loss, need for non-invasive ventilation using the bilevel positive airway pressure, gastrostomy, advanced respiratory life support, and tracheostomy ( Table 1 ).

**Table 1:** Baseline characteristics of the patients (N = 46)

Variable		
Sex	Male (n)	32
	Female (n)	14
Mean age (years)		55.8
Mean age at disease onset (years)		51.7
Distance from the center where the teleconsultations were performed (km)	Minimum	0
	Maximum	354
	Mean	115
Onset phenotype: n (%)	Bulbar	10 (22)
	Spinal	36 (78)
Clinical phenotype: n (%)	Classic	24 (52)
	Progressive bulbar palsy	5 (10)
	Progressive muscular atrophy	4 (9)
	Primary lateral sclerosis	1 (2)
	Flail arm	3 (7)
	Hemiplegic amyotrophic lateral sclerosis	2 (4)
	Pseudopolyneuritic amyotrophic lateral sclerosis	3 (7)
	Unknown	4 (9)
El Escorial classification: n (%)	Definite	14 (30)
	Probable	16 (35)
	Possible	9 (20)
	Unknown	7 (15)
King's Staging: n (%)	1	2 (4)
	2	5 (11)
	3	12 (26)
	4a	0
	4b	27 (59)
Amyotrophic Lateral Sclerosis Functional Rating Scale-Revised: n (%)	< 39	36 (78)
	≥ 39	10 (22)
Disease progression: n (%)	Fast (> 1)	7 (15)
	Intermediate (0.5 to 1.0)	19 (41)
	Slow (< 0.5)	20 (44)
Non-invasive mechanical ventilation: n (%)		25 (54)
Gastrostomy: n (%)		12 (26)
Tracheostomy: n (%)		5 (11)
Home-based care: n (%)		7 (15)

**Figure 1:**
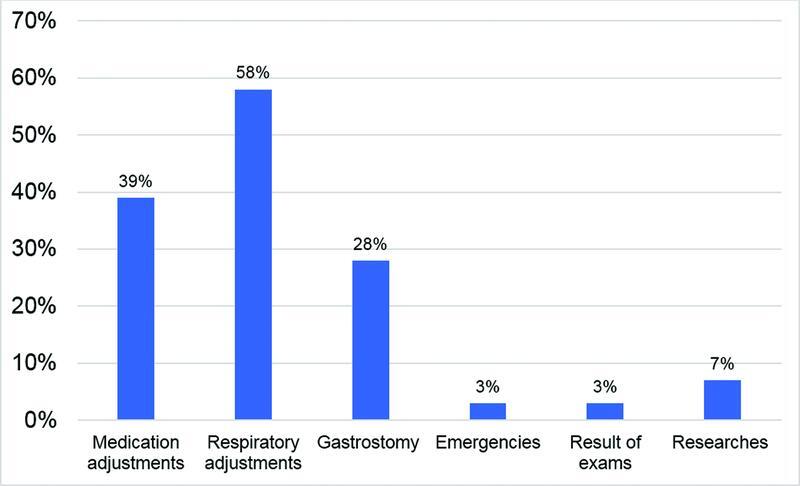
Topics addressed during the teleconsultations.

Respiratory adjustment (such as the use or adjustment of non-invasive ventilation, masks, manual insufflator, and mechanical insufflation-exsufflation) was the most addressed topic in the teleconsultations, followed by medication adjustments ( Figure 1 ).

Due to instability in the internet connection or difficulties accessing the Teleconsulta platform, some consultations were performed using the Google Meet platform. In case of limited internet access, patients were individually consulted through phone calls. The lack of limited internet access, computer or smartphone incompatibility with the platform, illiterate patients, difficulty in properly accessing the platform, and poorly-positioned smartphone cameras (which prevented the physician from seeing the patient, for example) were the most common problems.

A total of 33 caregivers ( Table 2 ) and 36 patients ( Table 3 ) answered the questionnaire on the level of satisfaction. Although the teleconsultations were well evaluated, patients and caregivers reported that the experience differed from the regular in-person consultation.

**Table 2:** Questionnaire on the level of satisfaction of caregivers (%)

	(1)	(2)	(3)	(4)	(5)
I was able to communicate properly with the healthcare professionals.	0	3	6	64	27
I was able to address my concerns with the healthcare professionals during teleconsultations.	0	0	6	67	27
The healthcare professionals provided recommendations to solve my concerns during the teleconsultation.	0	0	0	64	36
I had enough time to discuss my concerns with the healthcare professionals.	0	0	6	82	12
I was comfortable using the videoconference platform during the teleconsultation.	0	0	0	76	24
The teleconsultation provided a similar experience to my regular in-person consultation.	0	27	39	27	7
The teleconsultation was useful.	0	3	9	58	30
I am confident to use and operate the videoconference platform for teleconsultations.	0	3	12	61	24
The videoconference platform was easy to set up before the teleconsultation.	0	6	15	61	18
The time required to set up the videoconference platform for the teleconsultation was sufficient.	0	3	27	55	15
The videoconference platform was easy to use during the teleconsultation.	0	9	12	61	18
Overall, I was satisfied with the teleconsultation.	0	3	12	58	27
I would participate in teleconsultations again in the future.	0	3	3	64	30

Note: Scores – (1) strongly disagree; (2) disagree; (3) neither agree nor disagree; (4) agree; and (5) strongly agree.

**Table 3:** Questionnaire on the level of satisfaction of patients (%)

	(1)	(2)	(3)	(4)	(5)
I was able to properly communicate with the healthcare professionals.	0	6	0	61	33
I was able to address my concerns with the healthcare professionals during teleconsultations.	0	3	6	61	30
The healthcare professionals provided recommendations to solve my concerns during the teleconsultation.	0	0	0	72	28
I had enough time to discuss my concerns with the healthcare professionals.	0	3	3	61	33
I was comfortable using the videoconference platform during the teleconsultation.	0	0	6	64	30
The teleconsultation provided a similar experience to my regular in-person consultation.	0	25	25	39	11
The teleconsultation was useful.	0	0	0	64	36
I am confident to use and operate the videoconference platform for teleconsultations.	3	0	25	53	19
The videoconferencw platform was easy to set up before the teleconsultation.	3	8	28	44	17
The time required to set up the videoconference platform for the teleconsultation was sufficient.	0	0	22	61	17
The videoconference platform was easy to use during the teleconsultation.	0	0	17	56	25
Overall, I was satisfied with the teleconsultation.	0	0	3	53	44
I would participate in teleconsultations again in the future.	0	0	3	50	47

Note: Scores – (1) strongly disagree; (2) disagree; (3) neither agree nor disagree; (4) Agree; and (5) strongly agree.

## DISCUSSION

Patients with ALS and caregivers evaluated well the teleconsultations. They highlighted the convenience of receiving assistance and maintenance of multidisciplinary care at home. However, the patients and caregivers reported the lack of physical contact and difficulties with technology as negative issues. The teleconsultation promoted engagement among the multidisciplinary team to discuss and evaluate patient and exchange information with professionals the city of origin of the patient. Drug prescriptions and exam requests were delivered to a caregiver at the referral center one week after the teleconsultation.

Before the COVID-19 pandemic, a small portion of patients were submitted to telehealth, and patients, caregivers, and health professionals reported a perception of satisfaction and safety. 21 22 23 24 However, access to technology, a medical license to work only through teleconsultations, and the costs with the health insurance to establish the service were described as the main barriers. 25 The field of neurology also adopted telehealth, including for the treatment of neurodegenerative and neuromuscular diseases. 26 27 Telehealth services to patients with ALS increased during the COVID-19 pandemic, and many studies demonstrated its viability. 15 16 28 29

Teleconsultation benefits the follow-up and revaluations of patients with end-stage diseases, since they are not exposed to the hospital environment, and do not have to wait for consultations and suffer from fatigue, and have no need for transportation to a distant referral center. Some patients in the present study lived 354 km away from our center, which could represent a high cost for the family and public health system of the city. There have been reports in the literature of significant reductions in cost for patients, families, and the public health system due to teleconsultation. 30

Patients and caregivers reported the lack of privacy to discuss intimate issues or talk exclusively with the health professional as the main negative point. Another limitation was the lack of physical evaluation, which is relevant in cases in which the possible diagnosis requires regular physical revaluations to monitor new signs and symptoms. To minimize this limitation, the patients were asked to show and make some movements with their hands, arms, feet and legs so that the multidisciplinary team could better assess disease progression. In addition, patients were systematically asked about fasciculation and cramps.

Most patients in the present study had advanced ALS (that is, stage 4b on the King's Staging), 18 corroborating the data on ALSFRS-R < 39 (74%) and use of non-invasive ventilation (54%). Thus, data on the disease severity among patients with ALS in the present study was consistent. Teleconsultations are complex for advanced ALS due to several clinical and symptom demands (such as sleep and mood disorders, drooling, chronic pain, spasms, weight loss, and respiratory dysfunctions). 13 14 Therefore, respiratory and medication adjustments were the most addressed topics in the present study.

Clinical emergencies were often addressed through phone calls or text messages (depending on each case). In these moments, instructions and changes in clinical approaches were performed to avoid negative outcomes. Thus, the Teleconsulta platform recorded a small number of emergency approaches.

Patients and caregivers reported teleconsultations as positive and satisfactory Also, teleconsultation enabled multidisciplinary care with lower risks and costs regarding transportation to reference centers. Thus, teleconsultation should be prioritized for patients with advanced diseases to discuss their cases with caregivers and the care team from the patient's city.

The main limitation of the present study was the lack of a control group to compare the level of satisfaction between in-person consultations and teleconsultations.
